# KMR: knowledge-oriented medicine representation learning for drug–drug interaction and similarity computation

**DOI:** 10.1186/s13321-019-0342-y

**Published:** 2019-03-14

**Authors:** Ying Shen, Kaiqi Yuan, Min Yang, Buzhou Tang, Yaliang Li, Nan Du, Kai Lei

**Affiliations:** 1 The Shenzhen Key Lab for Information Centric Networking and Blockchain Techologies(ICNLab), School of Electronics and Computer Engineering, Peking University Shenzhen Graduate School, 518055 Shenzhen, People’s Republic of China; 2 SIAT, Chinese Academy of Sciences, 518055 Shenzhen, People’s Republic of China; 3 School of Computer Science and Technology, Harbin Institute of Technology (Shenzhen), Shenzhen, 518055 People’s Republic of China; 4 Tencent Medical AI Lab, Palo Alto, USA; 5 PCL Research Center of Networks and Communications, Peng Cheng Laboratory, Shenzhen, China

**Keywords:** Drug embeddings, Knowledge representation, Drug–drug interaction, Drug–drug similarity, Feature processing

## Abstract

Efficient representations of drugs provide important support for healthcare analytics, such as drug–drug interaction (DDI) prediction and drug–drug similarity (DDS) computation. However, incomplete annotated data and drug feature sparseness create substantial barriers for drug representation learning, making it difficult to accurately identify new drug properties prior to public release. To alleviate these deficiencies, we propose KMR, a knowledge-oriented feature-driven method which can learn drug related knowledge with an accurate representation. We conduct series of experiments on real-world medical datasets to demonstrate that KMR is capable of drug representation learning. KMR can support to discover meaningful DDI with an accuracy rate of 92.19%, demonstrating that techniques developed in KMR significantly improve the prediction quality for new drugs not seen at training. Experimental results also indicate that KMR can identify DDS with an accuracy rate of 88.7% by facilitating drug knowledge, outperforming existing state-of-the-art drug similarity measures.

## Introduction

Discovering proper representations of high dimensional concepts has received much attention lately due to its impressive performance in numerous natural language processing (NLP) tasks across multi-task learning [1], question answering [2], semantic textual similarity [3], sentiment analysis [4], and knowledge generative discovery [5]. Using various types of neural networks, high-dimensional data can be converted into continuous real-valued concept vectors that effectively capture their latent semantics from the data [6]. Despite the success of current studies, efficient drug representations are an important but challenging task for four reasons:

1. Impressive drug representation learning was achieved in domains where a complete dictionary or a knowledge base is available [7]. However, the number of drug is constantly growing but the medical dictionary or knowledge base updating procedure is slow. For new drugs, the lack of clinical data and application data is almost inevitable. To overcome this limitation, it is common for us to rely on carefully designed feature representations. Nevertheless, the feature selection and processing remains a challenge in real-world applications.
2. Drug-specific information, which plays a crucial role in learning drug representation and similarity or interaction metric, is yet to be well-researched. Drug concepts contain rich latent information that cannot be represented solely through pharmacology and drug catalog knowledge. For example, *clopidogrel* is an alternative medicine of *aspirin*, they are obviously more related than the pair of *clopidogrel* and *crestor* which are in the same cardiovascular drug category. However, without the drug description information, it is difficult to correctly identify the drug relation between them. Despite its usefulness, the application of drug description information in drug presentation is still under-explored [8].
3. The issues of complex and diverse terminology, relations, hierarchies and attributes in the medical field remain yet to be resolved. The existing computation measures of semantic similarity based on Knowledge base (KB) can be classified into path/depth-based similarity measures and corpus-based methods. However, path-based and depth-based similarity measures cannot adequately handle the computation between two concepts with the same path but different semantic similarity in the KG taxonomy [9], while Corpus-based methods are substantially dependent on the training corpus and susceptible to data sparseness and data noise [10].
4. The interactions between different drug features derived from various text and knowledge bases have received little attention in existing drug representation learning methods [11], which regard each feature as an independent item without any correlation to other features. This attribute independence assumption does not always work in medical scenarios because drug features usually have strong correlations with each other. For example, cannabis has various *physiological effects* on the human body. When exceeding the psychotropic threshold, users may experience *adverse side effects* such as anxiety and panic attacks. Therefore, the assumption of feature independence may affect its representation learning.

To alleviate these limitations, we propose a knowledge-oriented medicine representation learning method named KMR for drug–drug interaction prediction and similarity computation. In specific, we first learn the initial drug representation by processing the features with respect to pharmacology, drug class, and drug description information. Then, we develop an interactive learning schema within deep neural network to discover the interaction information among features. After learning the drug embeddings, we conduct experiments on a real-world dataset on the drug–drug interaction prediction and similarity computation. Experimental results demonstrate that, our method can effectively perform joint representation learning and obtain more informative knowledge representation, which significantly outperforms other baseline methods.

The main contributions of this paper can be summarized as follows:

- We propose a novel knowledge-oriented medicine representation learning model, which leverages the pharmacological features, drug class features and drug textual description features within neural network architecture to alleviate the limitation of incomplete or inaccurate public data sources;
- We develop an interactive learning scheme to emphasize respectively those features with rich information and exploit the interrelations among features based on the relevancy of various drug features;
- Experiments on real-world drug datasets demonstrate that compared with existing methods, KMR can effectively learn the drug representation, discover accurate drug–drug interaction with less training data, and identify drug–drug similarity for the drug substitution.

## Related work

Technically, the work in this paper relates to the representation learning of words, knowledge bases and textual information. Practically, our work is mainly related to the representation learning of drug. Related works are reviewed as follows.

*Representation learning of words* Learning pre-trained word embedding is a fundamental step in various NLP tasks. Word embedding is a distributed word representation which is typically induced using neural language models [12]. Several methods, e.g., Continuous bag-of-words (CBOW) and Skip-Gram [13], have been proposed for word embedding training, and have shown their power in NLP tasks.

There are many methods for learning word representations based on term-document, word-context, and pair-pattern matrices. For example, Turney et al. [14] presented a frequency-based method that follows the distribution hypothesis to conduct word representation via context learning. Mikolov et al. [15] learned high-quality distributed vector representations by predicting the word occurrences in a given context.

*Representation learning of knowledge bases* Representation learning of knowledge bases aims to embed both entities and relations into a low-dimensional space. Translation-based methods, including TransE [16], TransH [17], and TransR [18], have achieved the state-of-the-art performance by converting entities and relation into vectors and regarding each relation as one translation from head entity to tail entity. On the other hand, many studies have tried to use network embedding methods, e.g., Path Ranking Algorithm (PRA) [19], DeepWalk [20], node2vec [21], to reason over entities and relationships in knowledge base. The network embedding methods achieve the state-of-the-art performance of representation learning for knowledge bases, especially for those large-scale and sparse knowledge bases.

*Representation learning of textual information* Many studies have tried to automatically learn information from text using neural network models. For example, Socher et al. [22] introduced a recursive neural network (RNN) model to learn compositional vector representations for phrases and sentences of arbitrary syntactic type and length. Wang et al. [23] combined the convolutional neural networks (CNN) together with unsupervised feature learning to train highly-accurate text detector and character recognizer modules. Here attention mechanism can show its power. Many researchers have been interested in attention mechanism in neural networks and apply it to many areas such as machine translation [24], memory addressing [25] and image captioning [26].

Instead of learning the representations of different information separately, we develop a knowledge-oriented interactive learning architecture, which exploits the interactive information from input texts and knowledge bases to supervise the representation learning of words, structural and textual knowledge.

*Representation learning of drugs* Recently, some notable efforts have been made to design databases for drug representation learning and discovery. One well known example is DrugBank [27], a comprehensive resource that combines detailed drug (i.e. chemical) data with comprehensive drug target (i.e. protein) information. In addition to the DrugBank, a number of databases have also released including Therapeutic Target Database (TTD),^1^ Pharmacogenomics Knowledgebase (PharmGKB),^2^ and Kyoto Encyclopedia of Genes and Genomes (KEGG),^3^ Chemical Entities of Biological Interest (ChEBI)^4^ and PubChem.^5^ The on-line pharmaceutical encyclopedias such as RxList^6^ tend to offer detailed clinical information about many drugs but they were not designed to contain structural, chemical or physico-chemical information.

Many studies have demonstrated that it is possible to learn efficient representations of medical concept by improving the performance of medical predictive or classification models [28]. For example, Minarro et al. [29] learned the representations of medical terms by applying the word2vec deep learning toolkit to medical corpora to test its potential for improving the accessibility of medical knowledge. De Vine et al. [30] explored a variation of neural language models that can learn on concepts taken from structured ontologies and extracted from free text, for the task of measuring semantic similarity between medical concepts. Despite this progress, learning efficient representations of drug concepts, however, is still a relatively new territory and under-explored.

## Methodology

**Fig. 1:**
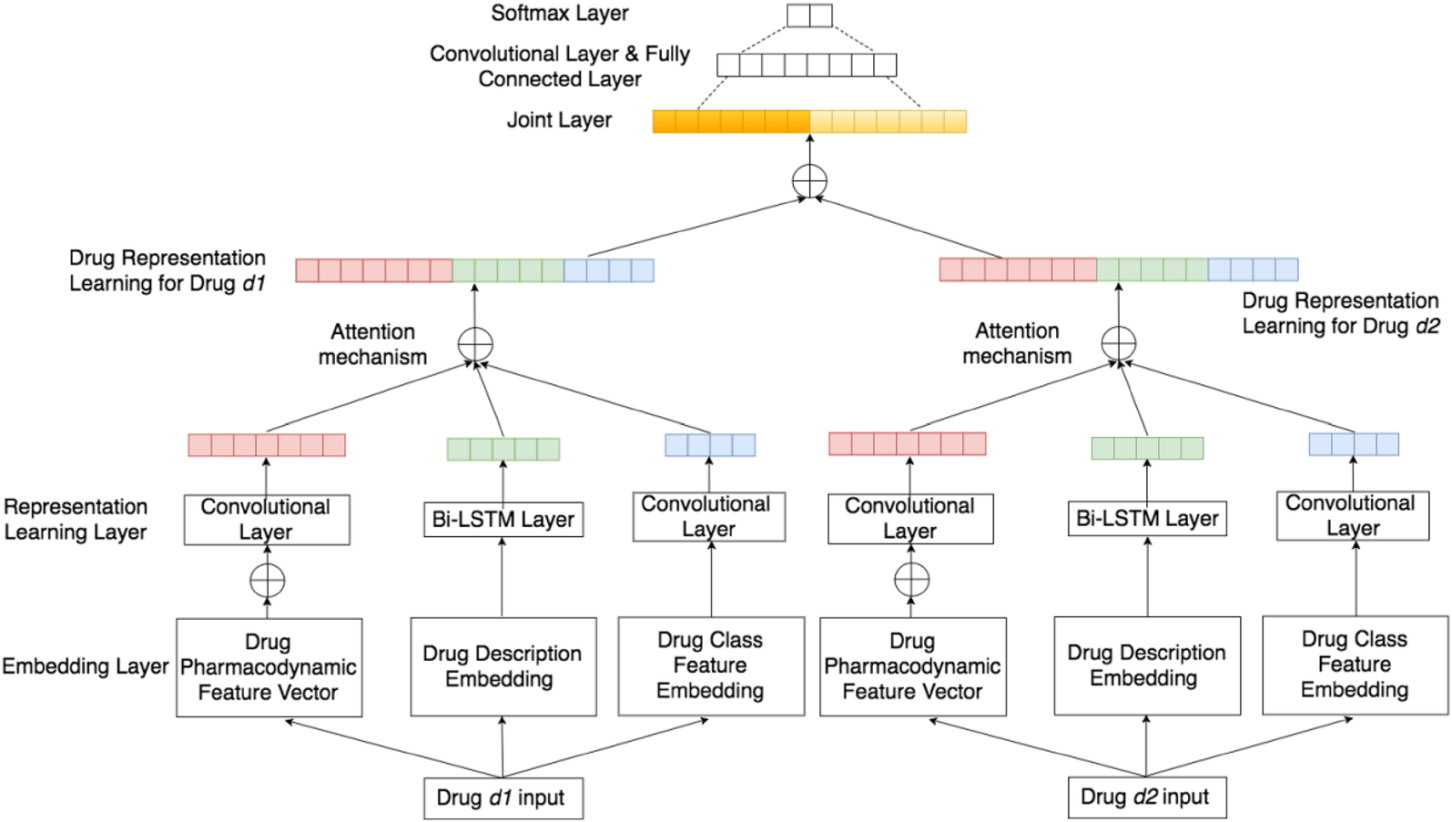
KMR for drug representation learning and drug-drug interaction prediction. Red, green, blue and yellow matrices denote pharmacological feature representations, drug textual description feature representations, drug class feature representations, and final knowledge-oriented drug representations, separately

We describe KMR in this section. Figure 1 illustrates the architecture of KMR.

Given a drug,

1. we employ neural network to learn the initial drug representation by simultaneously considering the features of the pharmacology, drug catalog, and drug description information.
2. Then an interactive learning scheme using attention mechanism is adopted to learn the interrelations among features.
3. Finally, there is a fully connected hidden layer to join all the features for the DDI binary classification or DDS computation.

**Fig. 2:**
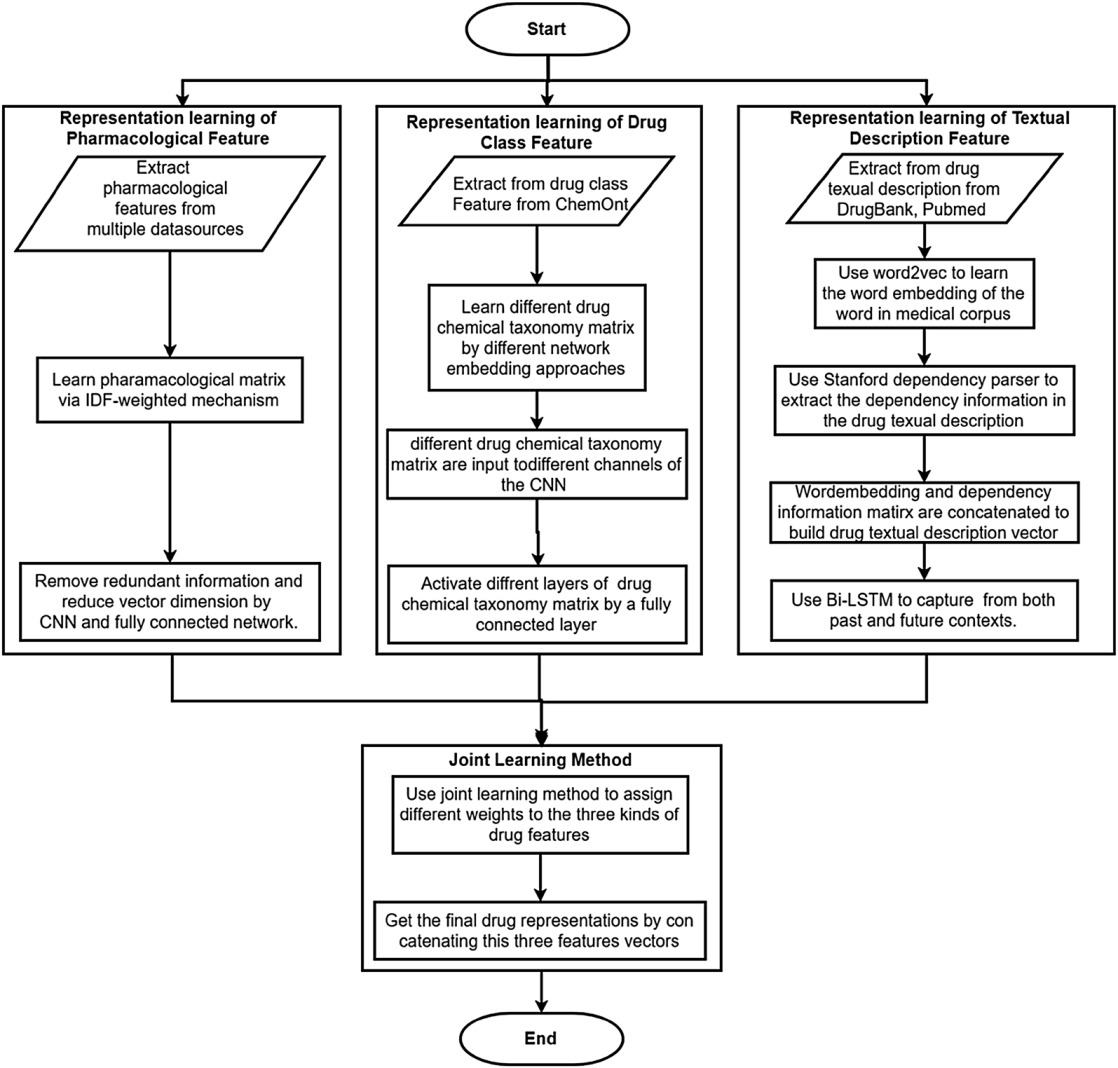
Algorithm flowchart of the entire KMR model

Algorithm flowchart of the entire KMR model is shown in Fig. 2:

### Datasets for medicine representation learning

*Knowledge bases* Drug side effect is collected from Side effect resource (SIDER)^7^ which contains 62,269 drug–side effect pairs and covers a total of 888 drugs and 1450 distinct side effects. Drug action is learned from and National Drug File-Reference Terminology (NDF-RT),^8^ which organizes the drug list into a formal representation for modeling drug characteristics including ingredients, mechanism of action, pharmacokinetics, and related diseases. The knowledge about the pharmaceutical formulation, physiological effects, drug targets and drug chemical structure are learned from DrugBank,^9^ which contains 11,680 drug entries including 2625 approved small molecule drugs, 1115 approved biotech (protein/peptide) drugs, 128 nutraceuticals and over 5504 experimental drugs. Drug class features can be extracted from ChemOnt^10^ which is a comprehensive and computable chemical taxonomy along with a fully annotated chemical ontology, allowing chemists and cheminformaticians to perform rapid and automated chemical classification.

*Text corpus* Given a drug, its textual description can be obtained from the “title” and “abstract”section of Pubmed,^11^ the “drug label information” section of DailyMed,^12^ and the “description”, “indication”, “pharmacodynamics”, “metabolism”, and “toxicity” section of DrugBank.^13^

*Synthetic dataset* The drug similarity is labelled by doctors ranging in [0, 1] from the perspective of clinical application. 0 indicates that there is no similarity between two antibiotics, while 1 implies that the two antibiotics are extremely similar. The adverse reactions, the patient’s past history and other factors are left out in this stage. To make drug pairs labeling more accurate, each pair is labeled by at least 3 doctors and the average is taken as the final result. The Pearson coefficient [36] between the scores issued by each doctor and the average score ranges from 82.7 to 86.4% while Spearman coefficient [37] ranges from 79.2 to 88.8%, both proving the reliability of doctors’ assessment.

### Representation learning of pharmacological feature

The representation learning of pharmacological feature aims to learn low-dimensional vector embeddings from various databases using deep learning models. Considering the application of a single dataset may cause the incompleteness of drug attribute, we employ multiple datasets to provide sufficient informative knowledge for the drug representation learning. The adopted datasets, features and their dimensions are summarized in Table 1.

**Table 1:** Datasets and features for the representation learning of pharmacological feature

Dataset	Pharmacological feature	Feature dimension
Sider	Side effect	4876
NDF-RT (National Drug File-Reference Terminology)	Drug action	626
	Physiological effects	1866
DrugBank	Pharmaceutical formulation	867
	Drug targets	3880
	Drug chemical structure	166

Accordingly, we consider the following features simultaneously:

*Side effect* Side effects indicates the secondary and usually adverse effect that occur when treatment goes beyond the desired effect. For example, the occurrence of *vomiting* and *hair loss* is an example of side effects that occur in addition to the therapeutic effects required to cancer treating.

Given a drug $d$, its side effect embedding $Sider\left( d \right)$ can be obtained by learning the side effect resource SIDER [31] using the IDF weighting method. The value of element ${\text{s}}$ of ${\text{Sider}}\left( {\text{d}} \right)$, denoted ${\text{Sider}}\left( {\text{d}} \right)\left[ {\text{s}} \right]$, is ${\text{IDF}}\left( {{\text{s}}, {\text{Drugs}}} \right)$ if it is one of the side effects of drug ${\text{d}}$, otherwise it is 0. $IDF\left( {s, Drugs} \right)$ can be calculated as:

1 $$IDF\left( {s, Drugs} \right) = log\left( {\left( {\left| {Drugs} \right| + 1} \right)/\left( {DF\left( {s, Drugs} \right) + 1} \right) } \right) ,$$

where $Drugs$ is the set of drugs, $s$ stands for a side effect, $DF\left( {s, Drugs} \right)$ is the number of drugs with side effect $s$.

*Drug action, pharmaceutical formulation, physiological effects and drug targets* Drugs are usually classified by their *drug actions*. For example, a *vasodilator*, prescribed to decrease the blood pressure, acts by dilating the blood vessels. *Pharmaceutical formulation* is the process in which different chemical substances, including the active drug, are combined to produce a final medicinal product. *Physiological effects* are those reactions resulting from some imbalance caused by taking a drug to the overall human system, or some specific part of it. A *drug target* is anything within a living organism to which some other entity (like an endogenous ligand or a drug) is directed and/or binds, resulting in a change in its behavior or function. Drug targets are most commonly proteins such as enzymes, ion channels, and receptors. The vectors of the aforementioned drug features are learned via the same IDF-weighted mechanism as mentioned in the previous paragraph.

**Fig. 3:**
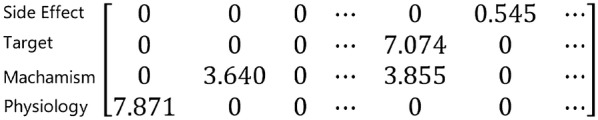
A snapshot of multi-dimensional weighted feature vector of an antibiotic: nitrofurantoin

Take “neostigmine” as an example (see Fig. 3). The drug target can be represented as a vector matrix of 584 × 326 dimensions, wherein the value of the 54th column is 5.2161745, and the other columns are all 0. For drug action, it can be represented as a vector matrix of the same dimensions, in which the values of column 152, column 157, column 187, column 222, column 251 and column 261 are 5.800606659291741, 5.395141551183577, 4.884315927417586, 5.800606659291741, 4.701994370623631, and 5.577463107977531 respectively. The other columns are all 0.

*Drug chemical structure* The chemical structure of a drug determines its physicochemical properties, further determines its ADME/Tox properties, and ultimately affects its pharmacological activity. We adopt PubChem Substructure Fingerprint^14^ that can generate a fingerprint, i.e., an ordered list of binary (1/0) bits, to learn the embeddings of drug chemical structure. Each bit represents a Boolean determination of the presence of PubChem features. For example, the bits (3, > = 32 H) and (11, > = 8 C) concerns the Hierarchic Element Counts, where “3” and “11” indicates the bit position, and “ > = 32 H” and “ > = 8 C” stands for the bit substructure. These bits test for the presence or count of individual chemical atoms represented by their atomic symbol. The presence of, for example, a type of ring system, atom pairing, atom environment (nearest neighbors), etc., in a chemical structure is determined by the same format (binary data).

The initial embeddings of all aforementioned features are concatenated to form the feature embeddings of drug $d_{i}$. To reduce the vector dimension, we input the feature representation $d_{i}$ to a Convolutional Neural Network (CNN). The fully connected layer of CNN model reduces the dimension of feature vectors from over 6000 dimensions to 500 dimensions, thereby improving the computation of embeddings.

### Representation learning of drug class feature

A drug class is a set of medications that have similar chemical structures, the same mechanism of action (i.e., bind to the same biological target), a related mode of action, and/or are used to treat the same disease [32]. To date, most attempts aimed at classifying and describing chemical compounds have been structure-based. This is largely because the bioactivity of a compound is influenced by its structure.

**Fig. 4:**
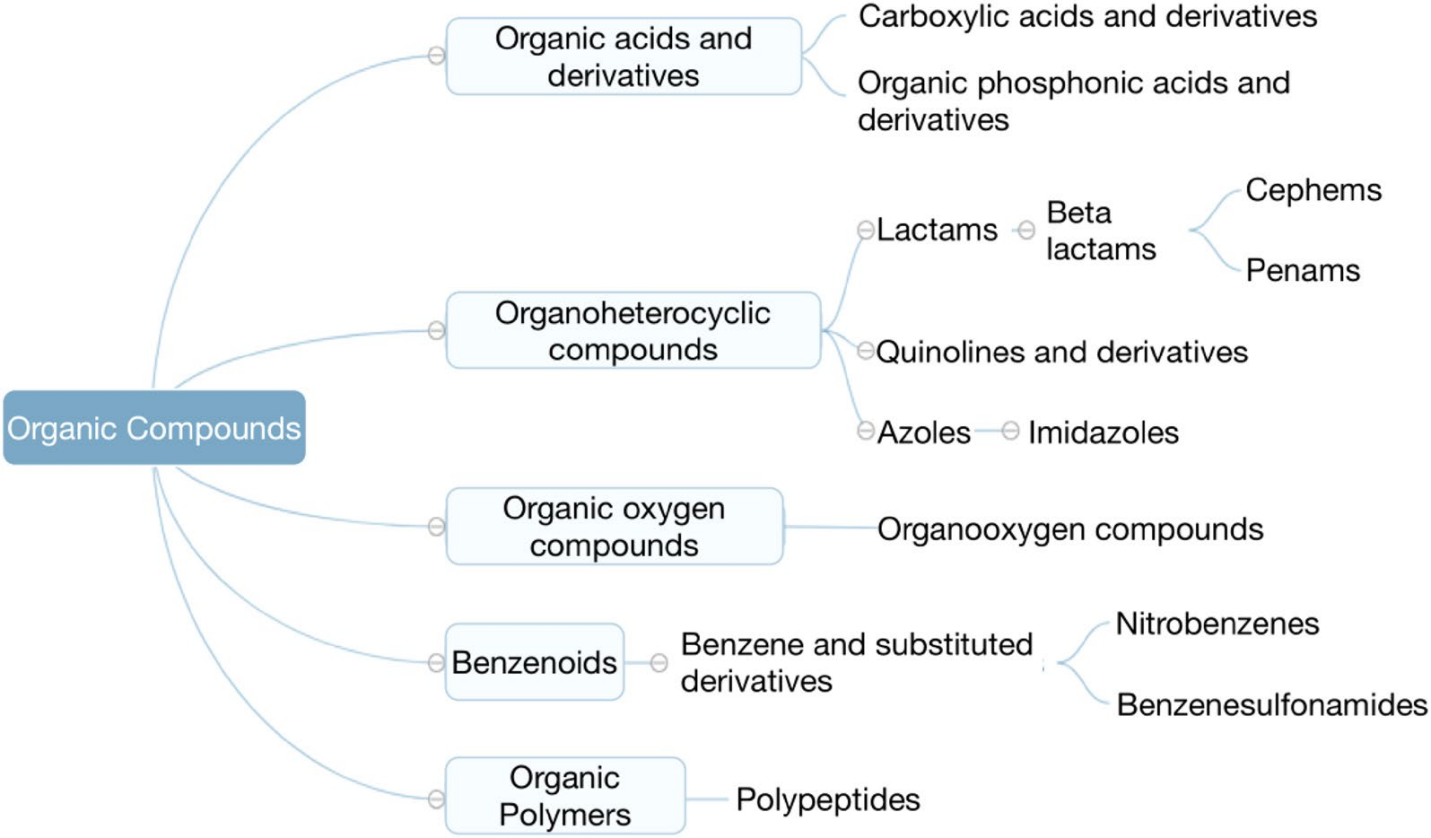
Part of an illustration of the drug taxonomy as a tree

Given the drug class taxonomy (see Fig. 4) referred from dictionary ChemOnt [33], a CNN is designed to learn the drug class representation from drug taxonomy.

**Fig. 5:**
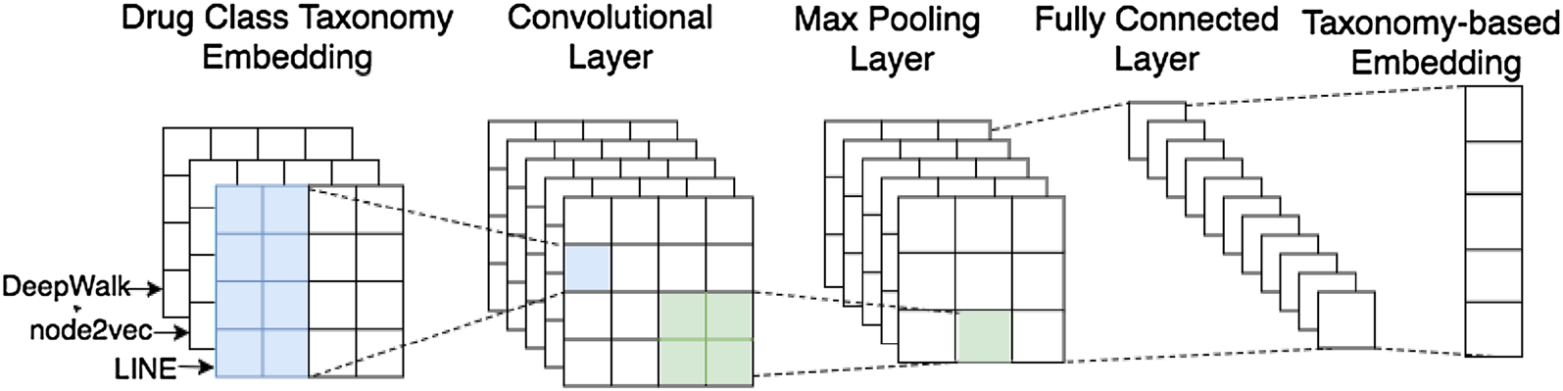
Processing of representation learning of drug class feature

*Input representation* Different network embedding approaches, i.e., DeepWalk, node2vec, and LINE, are adopted to learn drug chemical taxonomy. Generally, a convolution layer can have multiple input channels. The drug class embeddings ${\mathbf{D}} = \left\{ {{\mathbf{d}}_{1} ,{\mathbf{d}}_{2} , \ldots {\mathbf{d}}_{{\mathbf{n}}} } \right\}$, where ${\mathbf{d}}_{{\mathbf{i}}} \in {\mathbb{R}}^{k}$, $k$ is dimension of embeddings, learned by different network embedding approaches are input to different channels of the CNN, so as to make full use of all learned taxonomy information (see Fig. 5).

*Convolution layer* In the convolutional layer, the matrix of the drug class embeddings is convolved with filters of different sizes, generating a group of feature vectors. We first perform convolution operation over a sliding window then max-pooling to learn the stack vector of drug class embeddings ${\mathbf{D}}_{\varvec{n}}$, where ${\mathbf{D}}_{\varvec{n}} \in {\mathbb{R}}^{n}$.

*Fully connected layer* Finally, the vector obtained in the max pooling layer is fed to the fully connected softmax layer. Neurons in a fully connected layer have connections to all activations in the previous layer. In this study, the outputs of the fully connected layer is the embeddings of drug class feature.

### Representation learning of drug textual description feature

In this study, we incorporate dependency information into deep neural networks to extract entities and the relations between entities from drug textual description for the representation learning. For two medical entities (**en1** and **en2**) and a set of sentences (noted as *Sent*) containing both of them, the probability of the relation **r** between them is measured. For example, the sentence “**ALFENTA** can be administered in combination with other **CNS depressants**” indicates that ALFENTA and CNS depressants are positive correlation. Conversely, the sentence “Patients should be closely monitored for such adverse effects especially when **olanzapine** is added to **haloperidol** therapy” points out that the “olanzapine” and “haloperidol” are negative correlation. In this section, two parts about our model will be introduced.

#### Input representation

*Word embeddings* To feed training data to the neural network, the sentences we use are transformed into matrices. For a given sentence, it is represented by the embeddings of the words it consists of. The words are represented by real-valued vectors by looking up the pre-trained word embeddings.

*Dependency embeddings* The dependency feature used in the model are represented as vectors by looking up the corresponding embeddings. We choose the Stanford dependency parser^15^ to extract the dependency features. Dependency information can shorten the semantic distance between entities by organizing the whole sentence into a dependency tree [34]. Meanwhile, dependency features provide syntatic and semantic-level information, which can help the deep neural networks to learn with less training data [35].

Dependency information is obtained from the hierarchical structure of the dependency tree, including relative dependency features and dependency tags: *Relative dependency features* show the relation between the current word to the root of the tree or to the considered entities, and *dependency tags* imply the relationship between the current word and its parent node in the dependency tree.

*Relative dependency features*: Relative root feature implies the relation between current node and the root node. There are three types of relations here: the child node of the root, the root node itself, and others. Relative entity feature implies the relation between current node and entity1 and entity2. There are four types of relations: the child node of entity1/entity2, the parent node of entity1/entity2, entity node itself, and others.

*Dependency tags*: the tag of the current word to its parent node on the dependency tree.

**Fig. 6:**
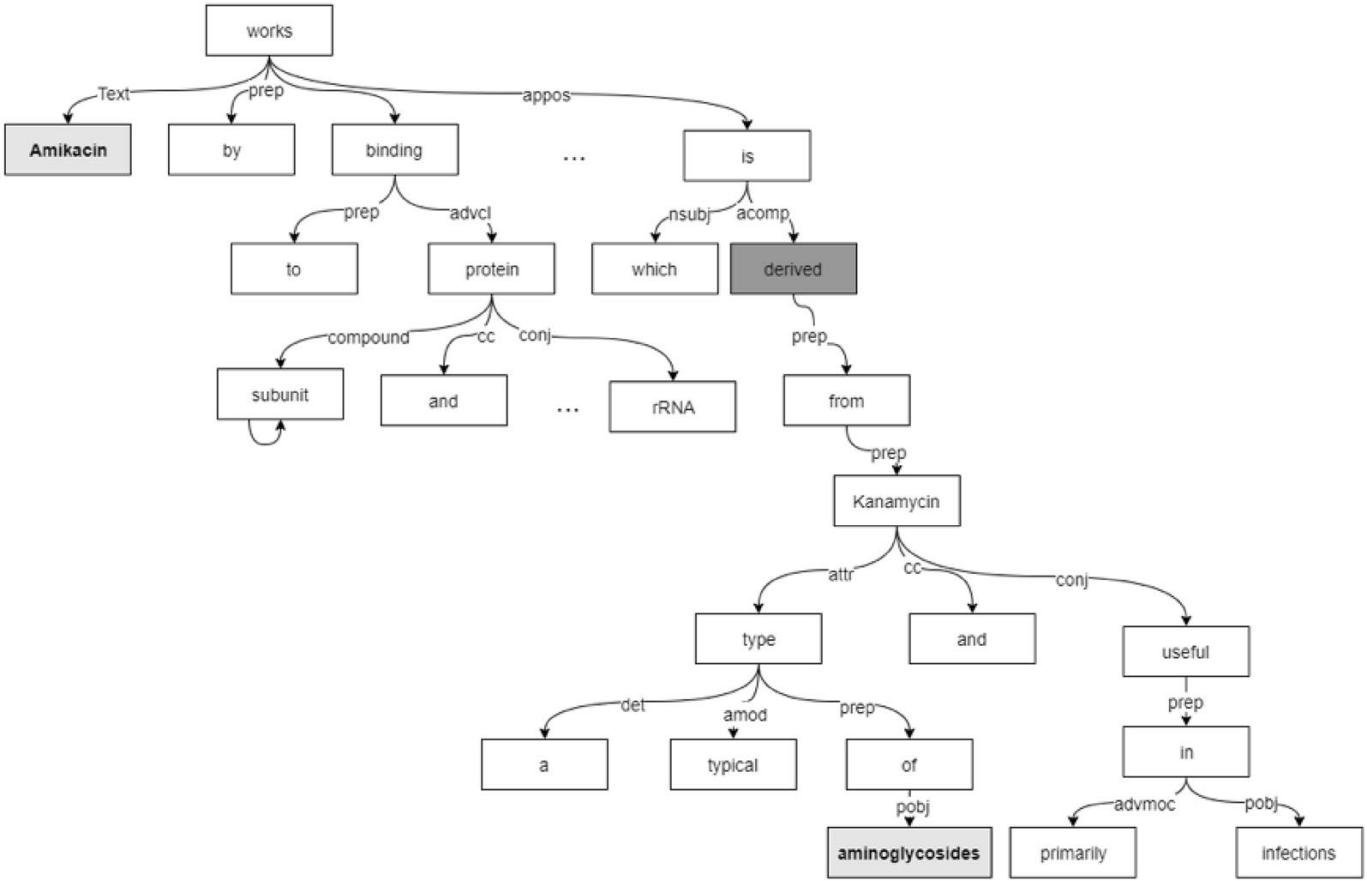
Dependency tree of the sentence containing “amikacin” (en1) and “aminoglycosides” (en2). The sentence is organized into a dependency tree. The linear sentence structure is transformed into a dense tree structure and the long-distance relationship between two entities in a sentence can be better captured

Figure 6 gives an example of a dependency tree structure. Due to the scale of the complete tree, only a part is shown here. Given a sentence “**Amikacin** works by binding to the bacterial 30S-subunit proteins and 16S rRNA,…*(30 words omitted here)*…, which is similar to other antibiotic **derived** from kanamycin. Kanamycin is a typical type of **aminoglycosides**…*(15 words omitted here)*…”, from the tree we can see that the word “derived” is the descendant node of entity1 (“amikacin”), and it is the ancestor node of entity2 (“aminoglycosides”). The distance between “amikacin” and “aminoglycosides” is thus shortened by a large margin compared to the original plain text.

Dependency features also need to be transformed into vectors to jointly use with word embeddings. Then the word embeddings $v_{i}^{w}$ and feature embeddings $v_{i}^{d}$ are concatenated to represent each word:

2 $$word_{i} = \left[ {v_{i}^{w} ,v_{i}^{d} } \right] .$$

#### Bi-LSTM and attention mechanisms

In this paper, we implement a Bi-directional LSTM network (Bi-LSTM) with combined word-level and sentence-level attention models. We employ Bi-LSTM model to capture the sequence information from both past and future contexts. We input the word vector sequence ${\text{W}}_{\text{l}} = \left[ {{\text{word}}_{1} ,{\text{word}}_{2} , \ldots {\text{word}}_{\text{j}} } \right]$ and the inverse word vector sequence ${\text{W}}_{{{\text{l}}_{\text{reverse}} }} = \left[ {{\text{word}}_{\text{j}} ,{\text{word}}_{{{\text{j}} - 1}} , \ldots {\text{word}}_{1} } \right]$ into the forward layer and backward layer of Bi-LSTM respectively. The output ${\text{hw}}_{\text{t}}$ at time step ${\text{t}}$, which combines the output of forward layer ${\text{hf}}_{\text{t}}$ and backward layer ${\text{hb}}_{\text{t}}$, can be calculated as:

3 $$hw_{t} = hf_{t} + hb_{t} .$$

In our model, sentence-level and word-level attention are complemented to de-emphasize the noisy samples and pay more attention to the useful information. Take sentence-level attention as an example. ${\text{a}}_{\text{i}}$ is the weight of a set of sentences containing a pair of entities, and it can be expressed as:

4 $$a_{i} = \frac{{{ \exp }\left( {e_{i} } \right)}}{{\mathop \sum \nolimits_{i} { \exp }\left( {e_{j} } \right)}} ,$$

where ${\text{e}}_{\text{i}}$ scores the relativity between the sentence and the predicted relation. Given a drug and its description, the outputs of the Bi-LSTM is the embeddings of drug textual description.

### Joint learning method

Given a drug and its corresponding features, i.e., pharmacological features, drug class features and drug textual description features, we apply attention mechanism to assign different weights according to the specific role each feature plays when interacting with other features. The representation of drug class feature $v_{c}$ are calculated as:

5 $$M_{w} = \tanh \left( {W_{sw} H_{w} } \right) ,$$

6 $$\alpha_{w} = {\text{softmax}}\left( {w_{w}^{T} M_{w} } \right) ,$$

7 $$v_{c} = H_{w} \alpha_{w}^{T} ,$$

where $M_{w} \in R^{dlxm}$ is a nonlinear mapping function, $W_{sw} \in R^{dlxdl}$ and $w_{w} \in R^{dl}$ are projection parameters, $\alpha_{w} \in R^{m}$ is the normalized attention. Other two types of features are processed by the same attention mechanism. Then these three type of feature embeddings are concatenated for the final knowledge-oriented drug representations.

For the DDI prediction task, there is a joint layer to join the final drug representations of drug 1 and drug 2. The outputs of the convolutional layer and fully connected layer then go through a softmax layer for binary classification:

8 $$y = softmax\left( {W_{o} pr + b_{o} } \right) ,$$

where each dimension of $y$ denotes the normalized probability of a certain relation, i.e., positive correlation or negative correlation, in accordance with the fully connected layer, ${\text{W}}_{\text{o}} \in {\text{R}}^{{2{\text{xdl}}}}$ is the projection matrix, and ${\text{b}}_{\text{o}} \in {\text{R}}^{2}$ is the offset vector.

For the DDS computation task, after joining the final drug representations of drug 1 and drug 2 through joint layer, we adopt a random forest regression model, which is an effective ensemble learning algorithm for regression task, to predict the similarity of a drug pair. Compared to other regression models, e.g., linear regression, logistic regression, etc., random forest is not very sensitive to missing data, which alleviates the impact from the incompleteness of drug attributes. Moreover, randomized sampling before bagging and the application of averaging can avoid overfitting and further improve the generalization ability.

### Experiment settings

For the CNN model, the kernel and the depth are set to 5 and 20 respectively. A Fully connected layer whose size is 500 is added after the CNN layer.

In the bidirectional long short-term memory (Bi-LSTM) implementation, we employ dropout on the output layer to prevent overfitting. We use ReLU activation function, take cross-entropy as loss function, and adopt AdaGrad as optimizer.

For both CNN and Bi-LSTM model, the learning rate and the dropout rate are set to 0.003 and 0.5 respectively. We train our models in batches with a size of 40. All other parameters are randomly initialized from [− 0.1,0.1]. The maximum length of sentence is set to 100.

For the base models, we follow exactly the same parameter settings as those in their original papers.

## Results and discussion

### Evaluation tasks: drug–drug interaction (DDI)

#### Drug–drug interaction (DDI) classification for different labeled prevalence

DDIs occur when two or more drugs are taken in combination and act on each other. To evaluate the proposed KMR method, we perform a retrospective evaluation using as the set of known DDIs pairs of interacting drugs presented in the 2017 version of DrugBank (V5.0.9). We adopt two baseline models for the experimental comparison:

- *Variational autoencoder (VAE)* An autoencoder is a type of artificial neural network used to learn efficient data coding in an unsupervised manner. VAE has become more widely used to learn a representation for a set of data, typically for the purpose of dimensionality reduction [38].
- *CNN model* the difference between KMR and its base CNN model is that the latter does not perform dimensionality reduction when learning the representation of pharmacological features.

**Fig. 7:**
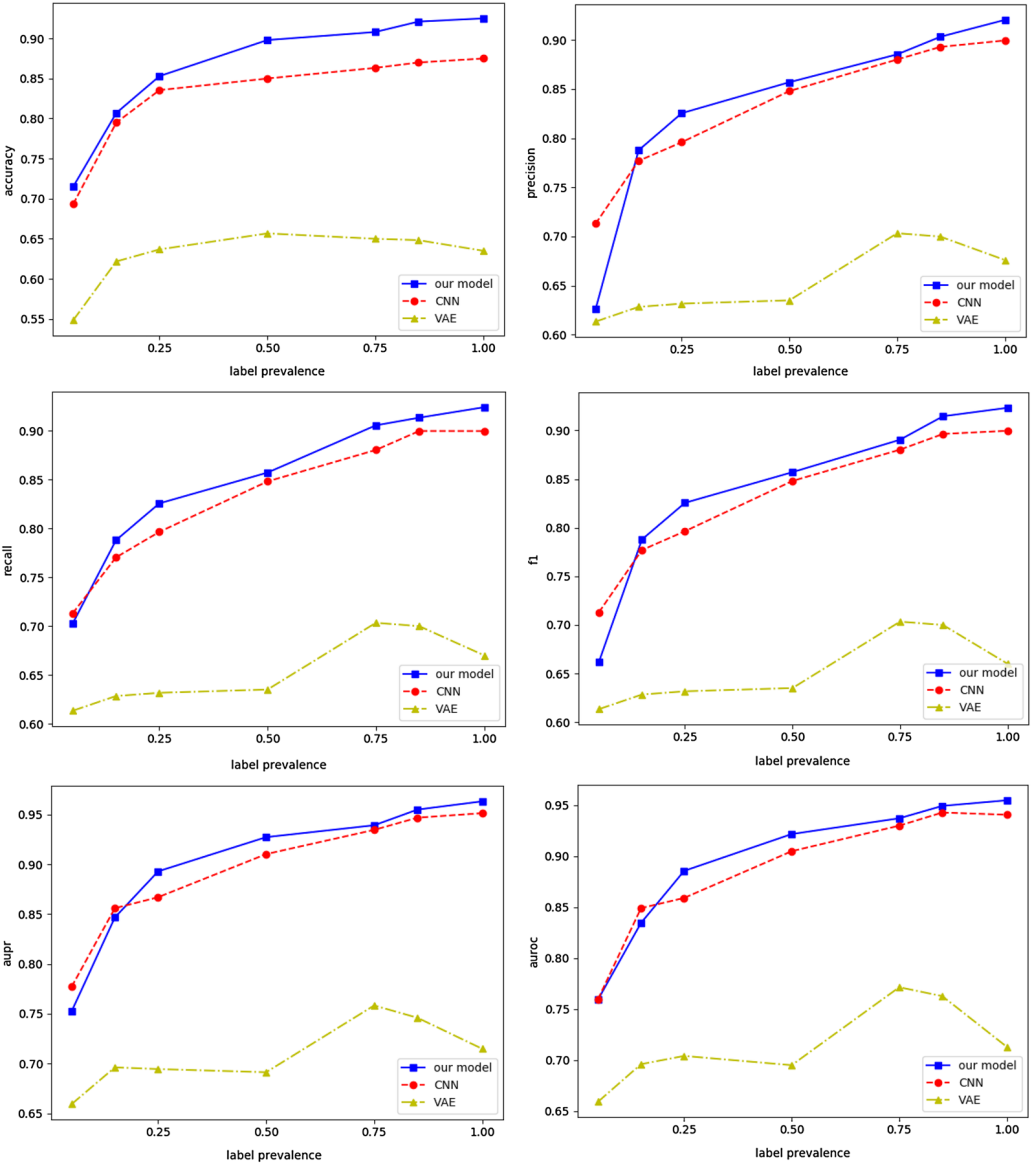
Comparison of different metrics for drug–drug interaction (DDI) prediction: accuracy, precision, recall, f1, aupr and auroc respectively. Using the same features with unbalanced training/validation data, KMR significantly outperforms the baselines

These baselines are a version of our system that uses the same input drug data and utilize the same set of aforementioned features. We randomly selected a fixed percentage (5%, 15%, 25%, 50%, 75%, 85%, and 100%) of training dataset for training, and compute the accuracy of the trained model in the testing dataset correspondingly. Regardless of the DDI prevalence used at training and validation, our approach significantly outperforms the baselines with respect to accuracy, recall, F1 score, and area under the precision-recall curve (AUPR) (see Fig. 7). For example, for a given 5% prevalence, our model achieves best accuracy (0.72 + 0.13) while CNN model cannot perform as well as our model (0.69 + 0.17).

In addition, as one may expect, training with higher prevalence actually improves the overall performance. For an assumed DDI prevalence at training ranging from 25 to 50%, the KMR accuracy rises from 0.85 to 0.90 when all features are used, while the accuracy improvements of the baseline models are very limited, demonstrating the ability of KMR to discover valid, but yet unknown drug–drug interactions in dataset even with limited labeled target data.

#### Drug–drug interaction (DDI) classification for newly developed drugs

We conduct a retrospective evaluation using the known DDIs and drug features in an earlier version of DrugBank (2016, V4.5) to predict the drug–drug interaction among newly developed drugs that presented in a more updated version of DrugBank (2017, V5.0.7). The performances of DDI prediction are measured by precision, recall, and F-score, receiver operating characteristic curve (ROC) and area under the precision-recall curve (AUPR), respectively.

For a given assumed DDI prevalence at training/validation and a DDI prevalence at testing, to get robust results and show the effectiveness of KMR model, six state-of-the-art baselines are adopted for comparison:

- *SVM* many state-of-the-art DDI extraction systems are based on support vector machines (SVM) with a large number of manually defined features [39].
- *FBK-irst* a multi-phase kernel based approach for drug–drug interaction detection and classification that exploits linguistic information [40].
- *CNN* a CNN model for DDI task consists of four layers: a look-up table layer, a convolutional layer, a max pooling layer, and a Softmax layer [41].
- *Att-BLSTM* an attention-based neural network model that uses RNN with LSTM units [42].
- *Tiresias* a large-scale similarity-based framework that uses utilizes two classes of features in a knowledge graph, i.e., local and global features, to predicts DDIs through link prediction [43].
- *LP-AllSim* an integrative label propagation framework to predict DDIs based on clinical side effects [44].

To analyze the effectiveness of our model, we also report the ablation test in terms of discarding the pharmacological feature (w/o pharmacology), drug class feature (w/o drug class) and drug textual description feature (w/o textual description), respectively.

With the same input (pharmacological feature, drug class feature, and drug textual description feature), different models with different representation learning and classification approaches yield different F-score and AUROC scores. There are multiple interesting observations from Table 2 as followings: (1) Compared with other state-of-the-art systems, our proposed KMR boosts the DDI prediction performance. It outperforms the current best system (CNN [41]) by 10% in F-score (see Table 2), mainly due to much higher precision. (2) Top performing systems in Table 2 (e.g., SVM [39], Tiresias [43],) are all performed based on various features such as features derived from medical resources. (3) Compared with the state-of-the-art SVM-based system, the advantage of our KMR lies in that it does not use any manually defined features generated by existing NLP toolkits. The features used in the KMR are automatically learned during training and may contain useful information beyond the manually defined features. Moreover, they effectively avoid errors caused by existing NLP toolkits. (4) Generally, all adopted features (i.e., pharmacological feature, drug class feature and textual description feature) contribute, and it makes larger performance boosting to DDI prediction. KMR substantially and consistently outperforms the ablation tests, demonstrating the necessity of simultaneous consideration of the proposed features.

**Table 2:** DDI Retrospective evaluation: training in an earlier version of DrugBank and testing in a more updated version of DrugBank. KMR correctly predicts up to 92.19% of the DDIs found after 2016

	Accuracy	Precision	Recall	F-score	AUROC	AUPR
FBK-irst	0.6533	0.6437	0.6867	0.6645	0.6807	0.7479
SVM	0.7867	0.7622	0.8333	0.7962	0.8844	0.8694
CNN	0.81	0.8039	0.82	0.8118	0.8892	0.8897
Att-BLSTM	0.7750	0.7749	0.7750	0.7750	0.8455	0.8486
Tiresias	0.80	0.7885	0.82	0.8039	0.8869	0.8861
LP-AllSim	0.77	0.7547	0.8	0.7767	0.8544	0.8600
KMR (our model)	*0.9219*	*0.9191*	*0.9191*	*0.9191*	*0.9512*	*0.9568*
w/o pharmacology	0.8571	0.8570	0.8571	0.8571	0.8571	0.8571
w/o drug class	0.8854	0.8854	0.8855	0.8854	0.8854	0.9391
w/o textual description	0.9033	0.9032	0.9033	0.9033	0.9373	0.9432

Results in italics identify the best values for the testing

#### Model analysis

For the three types of features proposed, when performing experiments on one type of feature separately, we will assume that the representation learning of the other two features are unchanged. Through the joint learning method described above, we obtain the feature vectors and apply them to the DDI prediction task, so as to verify the performance of feature embeddings.

A. Dimensionality reduction in representation learning of pharmacological feature

We conducted an experiment to verify whether the drug dimensionality reduction method used in pharmacological feature representation learning can improve the accuracy of DDI prediction. We choose several common dimensionality reduction methods as baselines:

- *concatenation* is to concatenate all pharmacological feature vectors, whose dimension is 5852.
- *trans_mat* is to multiply the pharmacological feature vectors by the mapping matrix, the resulting dimension is 100*6.
- *fully_conn* refers to the dimensionality reduction performed by a fully connected neural network. The vector dimension is reduced to 500.
- *our model* The pharmacological feature is processed by the convolutional neural network to obtain a vector with a dimension of 500.

**Fig. 8:**
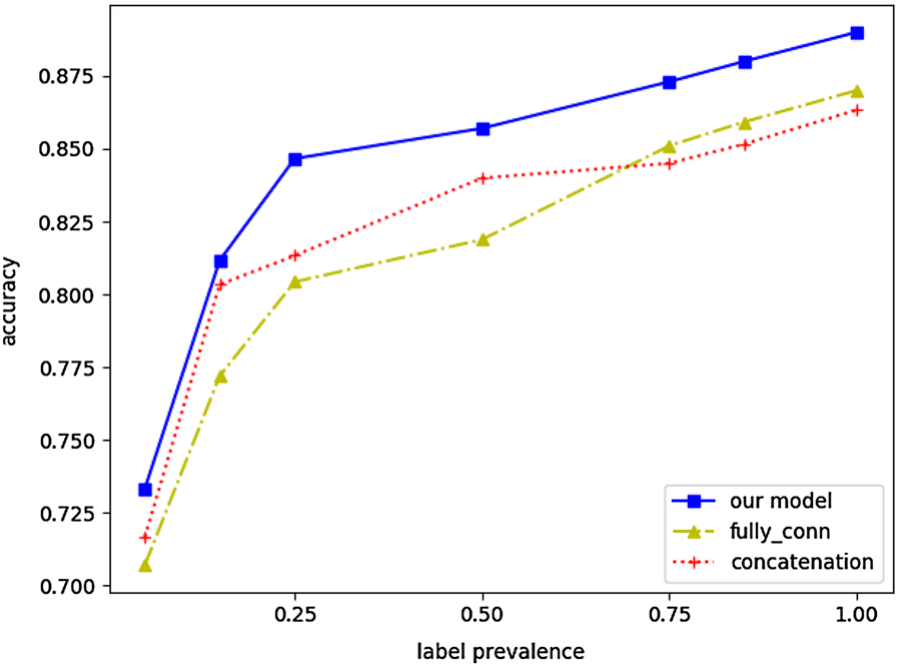
DDI prediction with different input (i.e., pharmacological feature vectors generated by different dimensionality reduction methods)

We verify the effectiveness of different dimensionality reduction methods by using their generated vectors in DDI prediction task. The accuracy is adopted as the evaluation metric. Figure 8 shows the performance evaluation for DDI prediction, whose input vectors are generated by different dimensionality reduction methods. Our model significantly outperforms all the baselines. We can also observe that concatenation is an easy and effective operation, which is robust and achieves a good performance on the DDI prediction task. Due to the poor classification effect (accuracy fluctuates around 0.50), the curve of trans_mat does not appear in the figure.

B. Performance of the representation learning of drug class feature

Table 3 reports the experimental results of DDI prediction with different drug class feature input generated by different network embedding methods. There are multiple interesting observations as follows: (1) The current translation-based methods, including TransE and PTransE, are the translation from head entity to tail entity. These methods are thus difficult to reasoning over paths between two entities. (2) Neural network models that performs multi-step relation inference in an embedding neural space, such as deepwalk, LINE, and node2vec, can well learn and present the drug taxonomy. (4) Our model inputs the drug class embeddings learned by deepwalk, LINE, and node2vec, respectively, to different channels of the CNN. We can observe that our method outperforms other methods. This improvement is benefit from the full use of all learned taxonomy information.

**Table 3:** Drug class feature embeddings learned by different network embedding methods

Model	Accuracy
TransE	78.5
PTransE	78.9
DeepWalk	80.8
LINE	80.7
Node2vec	80.7
Our model	*92.1*

Results in italics identify the best values for the testing

C. Performance of the representation learning of drug textual description feature

To demonstrate the effectiveness of textual description feature embeddings, we compare different textual embedding methods. The selected base models include:

1. *CNN* a convolutional neural network to extract lexical and sentence level features [45].
2. *PCNN* reducing the impact of noise and wrong label problems by employing Piecewise Max Pooling in convolutional neural network [46].
3. *BGRU* a Bidirectional GRU network with attention mechanism (BGRU-Att).^16^ Both (1)(2) are implemented with the sentence-level attention (-Att) to interpret the performance of our model.

**Fig. 9:**
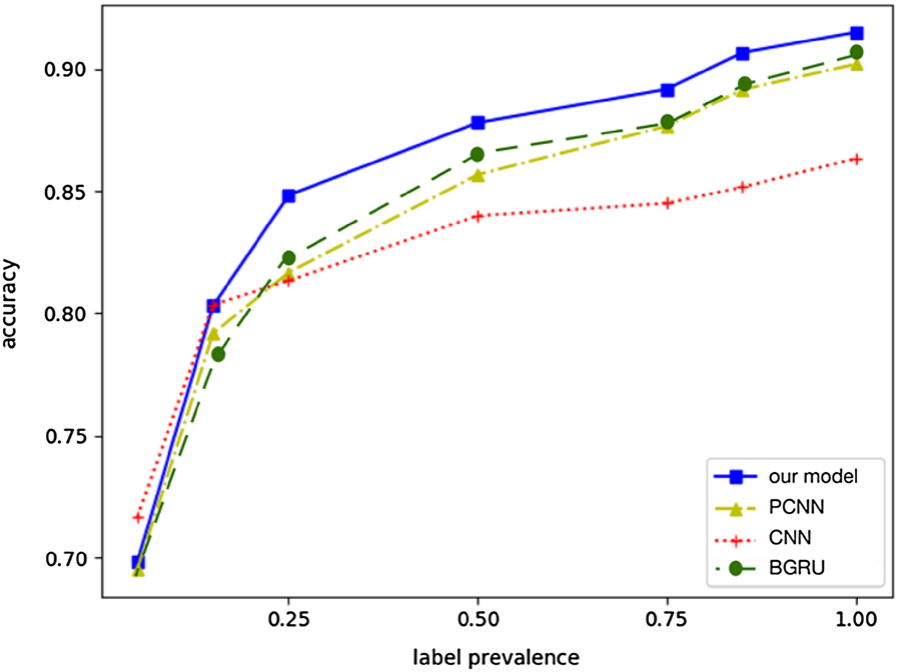
Accuracy of CNN, PCNN and our model in the DDI prediction task

Held-out evaluation is conducted and the results are shown in Fig. 9. In general, our model achieves the best performance. Dependency embedding improves the performance of our model. This is within our expectation since dependency information shortens the abstract distance (hops) in the dependency tree between source and target entities, as well as introduces structural and syntactic information to enrich overall sentence representation. The dependency embedding can reduce the semantic ambiguity thus alleviate the difficulty of relation extraction from cross-sentence long-text.

### Evaluation tasks: drug–drug similarity (DDS)

#### Drug–drug similarity (DDS) performance

Semantic similarity metrics in medicine has attracted substantial attention in recent years and its most promising application scenario is therapeutic substitution, also known as therapeutic interchange and drug substitution. Therapeutic substitution is the practice of replacing a patient’s prescription with chemically different drugs that are expected to have the same clinical effect [47].

To study drug substitution, we employ KMR to predict the similarity scores between cefoperazone and other antibiotics. Referring to [48], two antibiotics whose similarity scores over 0.85 can be replaced with each other under normal circumstances.

**Fig. 10:**
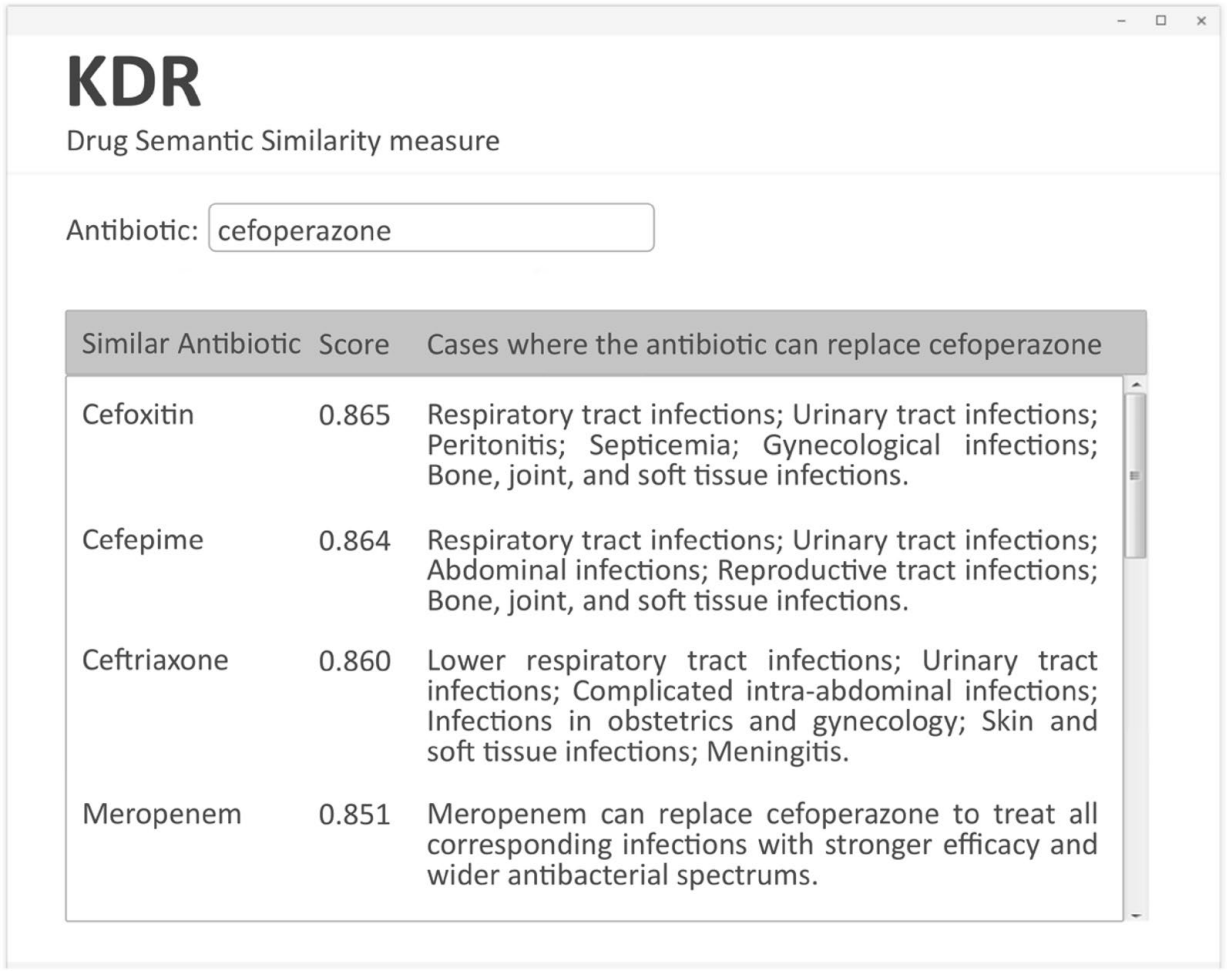
An example of drug similarity result provided by KMR

For the antibiotic cefoperazone, Fig. 10 presents antibiotics that are similar to it whose similarity score is over 0.85 and indicates the cases where they can replace each other. Take cefoperazone and ceftriaxone as an example. Ceftriaxone can replace cefoperazone in most cases except disease caused by a few bacteria such as Pseudomonas aeruginosa etc. In the absence of susceptibility testing, our method can help doctors to find the most appropriate drug substitution to treat most of Gram-negative bacteria infections, such as respiratory infection, pneumonia, and biliary infection.

#### Comparison with State-of-the-art similarity metrics

The experimental results on Drugbank are summarized in Table 4. Four state-of-the-art baselines are adopted for comparison: (1) The structure based measure GADES [10]; (2) The information content based measure Res [49]; (3) The Wpath method [50] considers both path information and information content; (4) The Hybrids method [51] which is based on Wpath takes medical properties into account to calculate the drug similarity. Pearson correlation coefficient and Spearman rank correlation coefficient are adopted to evaluate the correlation between doctors’ assessment and experiment results.

**Table 4:** DDS result on Drugbank (with ablation study)

	Pearson	Spearman
Res: Resnik et al. 2005	0.511	0.523
Hybrids: Hliaoutakis 2005	0.557	0.578
GADES: Traverso et al. 2016	0.652	0.602
Wpath: Zhu et al. 2017	0.750	0.703
KMR (our model)	*0.887*	*0.829*
W/o pharmacology	0.759	0.750
W/o drug class	0.778	0.711
W/o textual description	0.751	0.789

Results in italics identify the best values for the testing

We observe that KMR substantially and consistently outperforms the existing methods by a noticeable margin with respect to different correlations. For instance, on Drugbank, KMR improves by 13% on Spearman over these baselines. Experiment results reveal that on the analytics and assessments of KB semantic/structure information, domain specific features are important and need to be considered simultaneously.

In order to analyze the effectiveness of the different features of KMR, we also report the ablation test in terms of discarding the pharmacological feature (w/o pharmacology), drug class feature (w/o drug class) and drug textual description feature (w/o textual description), respectively. Generally, all factors contribute in similarity measure, and it makes larger performance boosting to measure medical semantic similarity. Even the basic system with pharmacological feature achieves competitive results with these strong baselines, which demonstrates the effectiveness of incorporating medical knowledge into measuring semantic similarity. It is proven that KB can introduce structural (drug class feature) and textual knowledge (drug textual description feature) to enrich overall knowledge representations, while medical knowledge can further enhance the knowledge representational learning of a specific domain.

## Conclusion

In this paper, we propose a knowledge-oriented method to capture the medical information, taxonomy information and semantic information of drugs, so as to explore the interaction and similarity between two drugs of interest.

In summary, our method is able to (1) learn medicine representation learning by capturing the medical information, taxonomy information and semantic information of drugs. (2) evaluate drug–drug interaction and drug–drug similarity. The KMR takes in various sources of drug-related data and knowledge as inputs, and provides DDI predictions as outputs. KMR is proved to be capable of dealing with drugs without any known interacting drugs. Experimental results on public dataset demonstrate that techniques developed in KMR significantly improve the prediction quality for new drugs not seen at training. The proposed method is reproducible and applicable to the drug representation learning and DDI computation. (3) process incomplete or inaccurate public data sources. We conduct experiments to show that a dataset with incomplete knowledge and structure sparseness can benefit from not only the application of various features but also the interactions between different features.

In the future, we will further utilize the attention scheme to effectively assemble the attentive information from different representational perspectives, so as to improve overall representational learning. In addition, we will perform an additional statistical significance analysis to rigorously demonstrate whether KMR’s improvement over other methods is statistically significant or not.

## Abbreviations

KMR: knowledge-oriented medicine representation learning
DDI: drug–drug interaction
DDS: drug–drug similarity
NLP: natural language processing
CBOW: continuous bag-of-words
PRA: path ranking algorithm
RNN: recursive neural network
CNN: convolutional neural networks
TTD: therapeutic target database
PharmGKB: pharmacogenomics knowledgebase
KEGG: Kyoto encyclopedia of genes and genomes
ChEBI: czhemical entities of biological interest
SIDER: side effect resource
NDF-RT: national drug file-reference terminology
Bi-LSTM: bidirectional long short-term memory
VAE: variational autoencoder
AUPR: area under the precision-recall curve
ROC: receiver operating characteristic curve
SVM: support vector machines

## Footnotes

1 bidd.nus.edu.sg/group/cjttd/.

2 https://www.pharmgkb.org/.

3 https://www.genome.jp/kegg/.

4 https://www.ebi.ac.uk/chebi/.

5 http://pubchem.ncbi.nlm.nih.gov/.

6 https://www.rxlist.com/.

7 http://sideeffects.embl.de.

8 https://www.nlm.nih.gov/research/umls/sourcereleasedocs/current/NDFRT/.

9 https://www.drugbank.ca/.

10 http://classyfire.wishartlab.com/.

11 https://www.ncbi.nlm.nih.gov/pubmed/.

12 https://dailymed.nlm.nih.gov/dailymed/drugInfo.cfm?setid=7d1950b4-3237-4512-bab3-4c7364bdd618.

13 https://www.drugbank.ca/drugs/DB00316.

14 https://pubchem.ncbi.nlm.nih.gov/.

15 https://nlp.stanford.edu/software/lex-parser.shtml.

16 https://github.com/thunlp/TensorFlow-NRE.
